# Investigation into the Active Substance of *Bacillus velezensis* TRM82367 for Killing *Aphis gossypii*

**DOI:** 10.3390/biology14111598

**Published:** 2025-11-15

**Authors:** Shiyu Wang, Xinyu Wang, Feng Wen, Zhanfeng Xia

**Affiliations:** State Key Laboratory Incubation Base for Conservation and Utilization of Bio-Resource in Tarim Basin, College of Life Science and Technology, Tarim University, Alar 843300, China; wsy0420yy@163.com (S.W.); 18399820103@163.com (X.W.); fengwen0306@163.com (F.W.)

**Keywords:** *Bacillus velezensis*, surfactin, *Aphis gossypii*, insecticidal activity

## Abstract

A total of 304 bacterial strains were isolated from the Taklamakan Desert. Through screening, strain TRM82367 exhibited potent insecticidal activity against cotton aphids (*Aphis gossypii*) and was identified as *Bacillus velezensis*. To explore the primary insecticidal active components of this strain, lipopeptide substances in the fermentation broth were extracted via hydrochloric acid precipitation. The main active components were analyzed using activity-guided fractionation combined with liquid chromatography–mass spectrometry (LC-MS). Our results demonstrated that *Bacillus velezensis* TRM82367 possessed strong lethal activity against cotton aphids. The crude extract obtained by means of hydrochloric acid precipitation and methanol dissolution showed high insecticidal efficacy. At concentrations of 100, 150, 200, 250, 300, 400, and 500 mg/L, the 48 h corrected mortality rates of cotton aphids were 25.14%, 38.05%, 50.09%, 53.03%, 57.96%, 74.68%, and 88.67%, respectively. The toxicity regression equation was Y = 2.47X − 5.72, with a median lethal concentration (LC_50_) of 207.616 mg/L and a 95% lethal concentration (LC_95_) of 1004.673 mg/L. After separation by an ODS chromatographic column and identification by means of mass spectrometry, the main active components were confirmed to be C_12_–C_16_ surfactin homologs.

## Simple Summary

The cotton aphid (*Aphis gossypii*) is a major pest that severely reduces cotton yield and quality by sucking sap, transmitting plant pathogens, and reproducing rapidly. Although chemical insecticides are widely used for control, they lead to resistance and environmental pollution, creating an urgent need for greener alternatives. In this study, we isolated a bacterial strain, TRM82367, from the extreme environment of the Taklimakan Desert, which exhibits strong activity against cotton aphids. The strain was identified as *Bacillus velezensis*. Genomic and chemical analyses revealed that it produces surfactin-like lipopeptides, which were confirmed to be the key active components causing high mortality in aphids. Our results suggest that *B. velezensis* TRM82367 and its surfactin compounds are promising candidates for developing bio-insecticides, especially suitable for cotton-growing regions in arid areas. This work supports the development of sustainable pest management strategies.

## 1. Introduction

The cotton aphid (*Aphis gossypii*), a small sap-sucking insect belonging to the order Hemiptera, suborder Sternorrhyncha, family Aphididae, and genus Aphis, is widely distributed across the globe [1]. Cotton is a major economic crop in China, with Xinjiang being its primary production region. In 2024, the national cotton planting area reached 2.8383 million hectares, of which Xinjiang accounted for 2.4479 million hectares [2]. Insect pests directly affect both the quality and yield of cotton; cotton aphid infestations, in particular, can lead to yield losses of 15–30% [3], resulting in significant economic damage. Cotton aphids harm plants through direct feeding, honeydew contamination, and transmission of plant viruses, leading to stunted growth, wilting, and the onset of various plant diseases, with severe cases causing plant death [4]. Moreover, aphids exhibit rapid reproduction, strong adaptability, and a reproductive period lasting 2–3 months [5], enabling them to thrive on a wide range of host plants.

Current aphid control strategies mainly include physical, biological, and chemical methods. Chemical control remains the most commonly used approach, employing insecticides such as imidacloprid, acetamiprid, thiamethoxam, and cypermethrin [6,7,8]. However, pesticide spraying is prone to drift [9], and frequent, high-volume applications readily lead to the development of pest resistance. In addition, pesticide residues negatively impact soil and water quality [10]. Consequently, there is an urgent market demand for green and novel agents for pest control.

With growing global emphasis on ecological protection and sustainable development, biopesticides have become a key research focus. The variety and application scope of such products continue to expand annually, with microbial pesticides showing the most rapid development. Approximately 70% of biopesticides worldwide are derived from bacteria; well-known examples such as spinosyns, avermectins, and *Bacillus thuringiensis* insecticidal crystal proteins have all been isolated from bacterial sources [11]. Ongoing research has revealed an increasing number of secondary metabolites obtained from *Bacillus* species, including peptides, siderophores, chitinases, and flavonoids [12]. *Bacillus velezensis* is a commonly studied bacterium known for its notable roles in biocontrol against plant pathogens and in promoting plant growth [13]. It has been reported to produce various secondary metabolites, such as surfactin, fengycin, iturin, and bacillomycin D [14]. Among these, surfactin has shown considerable potential in the pharmaceutical field, demonstrating antimicrobial, antiviral, and antitumor activities [15]; however, its application in pest control remains relatively underexplored.

This study aims to isolate a bacterial strain with high insecticidal activity against cotton aphids from the unique environment of the Taklimakan Desert and to elucidate the chemical nature of its active components. The taxonomic status of the strain will be determined via polyphasic classification, and bioassay-guided fractionation combined with LC–MS will be employed to identify the structures of the active constituents. This work intends to provide a promising candidate strain for the eco-friendly control of cotton aphids and to establish a foundation for the future development of novel bacterial insecticides with unique modes of action and minimal environmental impact.

## 2. Materials and Methods

### 2.1. Bacterial Strains

A total of 231 *Bacillus* and 73 *Streptomyces* strains were used in this study. All strains were previously isolated from soil samples collected in the Taklimakan Desert [16]. The sampling site was located at 36.9025° N, 81.2944° E, with an elevation of 1364 m. All isolates are preserved in the Microbial Culture Collection of Tarim University.

### 2.2. Test Materials

The cotton cultivar used in this study, Tahe 2, was purchased from an agricultural supply store in Alaer, Xinjiang Uygur Autonomous Region, China, in 2024. A growth substrate was prepared by mixing nutrient soil and vermiculite at a 5:2 ratio. Cotton plants were cultivated in an artificial climate chamber with regular watering to serve as a food source for aphid rearing.

In June 2025, cotton aphids were collected from the experimental field of Tarim University and subsequently maintained in rearing cages (50 × 50 × 65 cm) placed in the artificial climate chamber. Fresh cotton plants were provided as host material for continuous culture. The rearing conditions were set at 25 °C, 50% relative humidity, and a photoperiod of 16 h:8 h (light–dark), thereby supplying a stable source of aphids for the experiments.

### 2.3. Screening and Identification of Strains

The tested strains were subjected to scale-up cultivation via shake-flask fermentation. *Bacillus* strains were inoculated into Luria-Bertani (LB) medium and cultured at 30 °C with shaking at 120 rpm for 48 h, while *Streptomyces* strains were grown in Gauze’s No. 1 medium under the same conditions for 5–7 days. The fermentation broth was centrifuged, and the supernatant was collected for insecticidal activity screening using a spray method [17].

The specific operation was as follows: The supernatant of each test strain was uniformly sprayed on the cotton leaf surfaces with aphids. After standing for 5 min, 60 cotton aphids of uniform size were selected and placed into a 9 cm Petri dish. The bottom of the Petri dish was lined with filter paper moistened with sterile water to maintain humidity. Each test group was set with 3 replicates. The mortality rate was recorded after 48 h, and the corrected mortality rate was calculated. The negative control consisted of sterile culture medium without bacterial inoculation, and a 20% imidacloprid suspension was used as the positive control.

Mortality (%) = (Number of dead insects/Total number of treated insects) × 100(1)

Corrected mortality rate (%) = [(Observed mortality rate − Blank control mortality rate)/(1 − Blank control mortality rate)] × 100(2)

### 2.4. Species Identification of the Strain

#### 2.4.1. Morphological Observation

The bacterial strain exhibiting strong aphicidal activity was streaked onto LB agar plates and incubated at 30 °C for 48 h to observe colonial characteristics such as color and shape. For cellular morphology, the strain was cultured in liquid LB medium under the same conditions. Bacterial cells were collected by centrifugation, followed by drying and gold coating, and then examined using scanning electron microscopy (FE-SEM, Apreo S, Thermo Fisher Scientific, Waltham, MA, USA).

#### 2.4.2. Physiological and Biochemical Characterization

Physiological and biochemical properties of the target strain were determined following the methods described in The Manual for Identification of Common Bacterial Systems [18].

#### 2.4.3. Molecular Biological Identification

Genomic DNA was extracted using the CTAB-SDS method [19]. The 16S rRNA gene was amplified by PCR with universal primers 27F and 1492R. The PCR products were sequenced by Sangon Biotech Co., Ltd. (Shanghai, China). The resulting sequences were assembled using SeqMan Pro V7.1.0 software and compared for similarity against the EzBioCloud database (https://www.ezbiocloud.net/, accessed on 26 March 2025) to preliminarily determine the microbial species. A phylogenetic tree was constructed with MEGA software (version 7.0.26) using the Neighbor-Joining method. The phylogenetic position and relationships with closely related strains were analyzed to confirm the taxonomic status of the isolate.

### 2.5. Whole-Genome Sequencing, Assembly, and Annotation

A single colony of the target strain was inoculated into liquid LB medium and cultured with shaking at 30 °C and 120 rpm for 48 h. Bacterial cells were then harvested by centrifugation at 10,000 rpm for 5 min at 4 °C. Genomic DNA extraction and sequencing were performed by Shanghai Personal Biotechnology Co., Ltd. (Shanghai, China). Paired-end sequencing (2 × 150 bp) was conducted on the Illumina NovaSeq platform using a library with an insert size of approximately 400 bp.

Raw sequencing reads were quality-controlled using fastp to remove adapter sequences, low-quality bases (Q < 20), and short reads (<50 bp), resulting in high-quality clean reads. De novo assembly was performed from the clean reads using SPAdes. Scaffolds with a length ≥ 500 bp and an average sequencing depth ≥ 10× were retained as the initial assembly, which was subsequently polished at the base level using Pilon to yield the final high-quality scaffold sequences.

The completeness and contamination of the assembled genome were assessed with CheckM. Taxonomic classification was confirmed by two approaches: (1) BLASTN (v2.16.0) alignment of the assembly against the NCBI NT database; and (2) calculation of the Average Nucleotide Identity (ANI) using the Genome Taxonomy Database (GTDB) for further taxonomic assignment.

The genome annotation pipeline included: prediction of protein-coding genes (CDSs) with GeneMarkS; identification of tRNA and rRNA genes using tRNAscan-SE and Barrnap, respectively; and detection of other non-coding RNAs (ncRNAs) by alignment against the Rfam database. Protein-coding genes were functionally annotated using eggnog-mapper. Additionally, genomic islands were predicted with IslandViewer 4, prophage regions were identified using PhiSpy (v4.2.6), and biosynthetic gene clusters for secondary metabolites were predicted via the antiSMASH platform (https://antismash.secondarymetabolites.org/#!/start, accessed on 14 June 2025).

### 2.6. Isolation and Extraction of Lipopeptides

Based on the genomic prediction of secondary metabolites, the strain with cotton aphid-killing activity was subjected to shake flask fermentation, and lipopeptides were extracted using the hydrochloric acid precipitation method. The fermentation broth was centrifuged at 10,000 rpm for 10 min to collect the supernatant, which was adjusted to pH 2.0 with 6 mol/L hydrochloric acid and allowed to precipitate at 4 °C for 12 h.

The precipitate was collected by centrifugation at 12,000 rpm for 5 min, washed 2–3 times with deionized water (pH 2.0), and freeze-dried to obtain the crude hydrochloric acid precipitate [20]. The crude extract was fully dissolved in methanol and concentrated using a rotary evaporator to obtain an extract for subsequent use.

### 2.7. Insecticidal Activity Assay of Crude Extracts Against Cotton Aphids

A total of 0.1 g of the lipopeptide crude extract (paste) was weighed and dissolved in 10 mL of deionized water containing 0.5% DMSO. The mixture was vortexed to prepare a 10,000 mg/L stock solution (with a final DMSO concentration of 0.5% to avoid toxic interference with cotton aphids). Using deionized water containing 0.5% Tween 80 as the diluent, the stock solution was serially diluted to seven concentrations: 100, 150, 200, 250, 300, 400, and 500 mg/L. Each concentration was set with 3 replicates. Deionized water containing 0.5% Tween 80 was used as the blank control, and a 20% imidacloprid suspension was used as the positive control.

The aphid-killing activity was determined using the spray method: Cotton leaves infested with cotton aphids of uniform instar were uniformly sprayed with each concentration of the test solution. After standing for 5 min, 60 healthy cotton aphids were selected per replicate and placed in 9 cm Petri dishes lined with moist filter paper. The aphids were cultured at 25 °C with 50% humidity under a photoperiod of L/D = 16 h:8 h. After 48 h, the number of dead aphids was recorded, and the corrected mortality rate was calculated.

The probit regression method (IBM SPSS Statistics 27) was used to analyze the regression relationship between the logarithmic concentration and the corrected mortality rate (probit value). A toxicity equation was established, and LC_50_ and LC_95_ were calculated to evaluate the insecticidal activity of the lipopeptide crude extract against cotton aphids.

### 2.8. Identification of Cotton Aphid-Killing Active Substances

The above extract was separated using an ODS chromatographic column. Fractions exhibiting cotton aphid-killing activity were identified via insecticidal activity assays, and the types of lipopeptide compounds in the fermentation broth of the active strain were analyzed using liquid chromatography–mass spectrometry (LC-MS).

Liquid chromatography conditions: A Welchrom-C18 column (4.6 mm × 250 mm, 5 μm) was used with a column temperature of 40 °C and a flow rate of 1 mL/min. Mobile phase A was acetonitrile, and mobile phase B was ultrapure water. The injection volume was 10 μL. The elution program was as follows: 10% A at 0 min, linear gradient to 100% A at 40 min.

Mass spectrometry conditions: Mass spectrometer: Thermo Scientific LTQ Orbitrap XL (Thermo Fisher Scientific, Waltham, MA, USA); ion source: HESI; sheath gas flow rate: 40 mL/min; auxiliary gas flow rate: 10 mL/min; spray voltage: 3.0 kV (positive ion mode) and 2.5 kV (negative ion mode); capillary temperature: 300 °C; S-lens: 50; scanning mode: positive ion fullms-ddms2 top10. Scanning parameters: primary scan—resolution 70,000, mass range 134–2000 *m*/*z*; secondary scan—resolution 17,500, starting ion 50 *m*/*z*.

### 2.9. Statistical Analysis of Data

For strain screening, DPS V9.01 software was used to perform a significance test of differences via Duncan’s new multiple range test (*p* < 0.05). IBM SPSS Statistics 27 software was employed for statistical analysis, where the probit regression method was applied to calculate the toxicity regression equation and LC_50_. Microsoft Excel 2021 was used for data compilation, and Origin 2021 software was utilized for graph generation.

## 3. Results

### 3.1. Screening of Bacterial Strains with High Aphicidal Activity Against Cotton Aphids

Following the method described in Section 2.3, a total of 304 bacterial strains (231 *Bacillus* and 73 *Streptomyces* isolates) were screened for their aphicidal activity. Strains exhibiting a corrected mortality rate of ≥50% were selected for secondary screening, which identified four isolates demonstrating consistent insecticidal efficacy (Table 1). Strain TRM82367, which showed the highest activity against cotton aphids, was chosen for further investigation.

### 3.2. Taxonomic Identification of Strain TRM82367

#### 3.2.1. Morphological Observation

**Figure 1:**
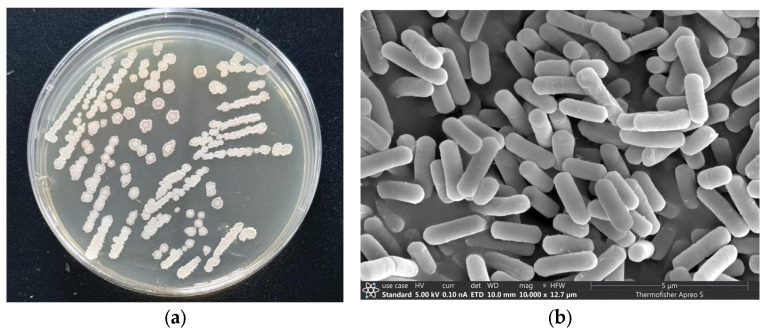
Morphological characteristics of strain TRM82367. (**a**) Colonial morphology on an LB agar plate after 48 h of incubation at 30 °C; (**b**) cellular morphology observed under scanning electron microscopy (SEM).

After 48 h of static incubation at 30 °C on LB agar plates, colonies of strain TRM82367 exhibited a regular circular form with a diameter ranging from 2.16 to 4.27 mm. The colonies appeared off-white in color, displayed a slightly raised profile, and were characterized by irregular serrated margins and a dry, wrinkled surface. Notably, the colonies did not form filaments when touched (Figure 1a). As shown in Figure 1b, scanning electron microscopy (SEM) at 10,000× magnification revealed that the cells were uniform, straight rods with rounded ends and smooth surfaces. No flagella or pili were visibly detected under the observed conditions.

#### 3.2.2. Physiological and Biochemical Characteristics

Strain TRM82367 was Gram-positive and exhibited positive oxidase and catalase activities. The strain utilized glucose and sucrose as carbon sources, but did not utilize Tween-20, Tween-60, Tween-80, citrate, galactose, or melibiose (Table 2). These physiological and biochemical characteristics are consistent with those of *Bacillus velezensis* CR-502 [21]. Based on its morphological, physiological, and biochemical profiles, strain TRM82367 was preliminarily identified as *Bacillus velezensis*.

#### 3.2.3. Molecular Identification of Strain TRM82367 Based on 16S rRNA Gene Sequence

**Figure 2:**
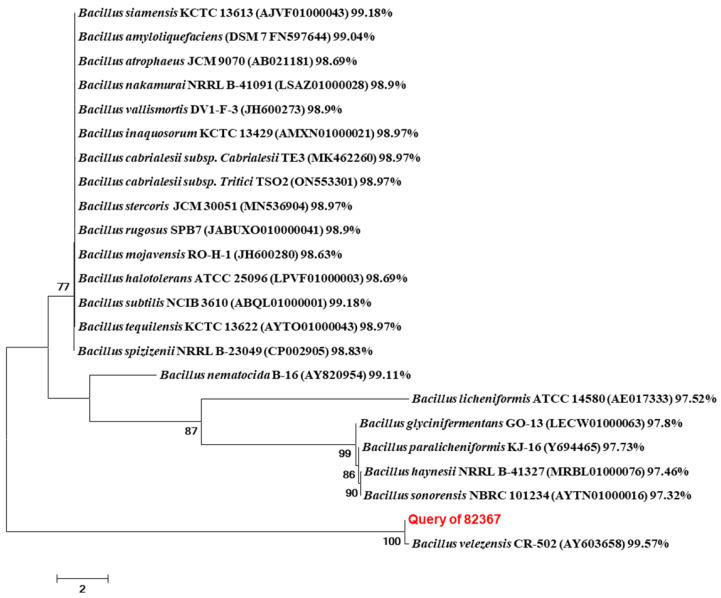
Phylogenetic tree of strain TRM82367 based on 16S rRNA gene sequences.

The 16S rRNA gene sequence of strain TRM82367 was deposited in the GenBank database under the accession number PX464590. Comparative analysis using the EzBioCloud database revealed that the 16S rRNA gene sequence of TRM82367 shares 99.57% similarity with that of *Bacillus velezensis* CR-502. A phylogenetic tree was constructed using the Neighbor-Joining method, incorporating 16S rRNA gene sequences of closely related type strains. As shown in Figure 2, strain TRM82367 forms a stable clade with *Bacillus velezensis* CR-502, supported by a high bootstrap value. These molecular results, consistent with the morphological and physiological data, confirm the identity of strain TRM82367 as *Bacillus velezensis*.

### 3.3. Genome Sequencing Results and Analysis of TRM82367

#### 3.3.1. Genome Assembly and Annotation

Gene prediction performed with GeneMarkS for strain TRM82367 identified a total of 3913 protein-coding genes, with a combined length of 3,453,788 bp. These genes accounted for 88.86% of the total genome length, with an average gene length of 883 bp. Functional annotation results indicated a genome with substantial metabolic potential.

Within the Clusters of Orthologous Groups (COG) classification, 3659 genes were assigned to 21 functional categories. The categories “Amino acid transport and metabolism”, “Carbohydrate transport and metabolism”, and “Biosynthesis, transport, and catabolism of secondary metabolites” accounted for the highest proportions of genes. This metabolic diversity was further supported by KEGG pathway annotation, in which 4011 genes were mapped to 1899 distinct pathways. Gene Ontology (GO) annotation assigned 7172 functional terms, with the majority being enriched in fundamental categories such as “Membrane” and “Catalytic activity”.

In terms of non-coding RNAs, a total of 84 ncRNA genes were identified, including 69 tRNAs. A complete ribosomal RNA operon was detected using Barrnap. Further comparison with the Rfam 14.9 database did not reveal other types of non-coding RNAs such as sRNAs, indicating that the strain’s non-coding RNA composition is predominantly comprised of fundamental types.

Analysis of genome plasticity revealed the genetic basis underlying its potential adaptability. Using IslandViewer 4, 21 genomic islands were identified, harboring multiple genes associated with antibiotic resistance and toxin synthesis. Additionally, 21 prophage regions, ranging in length from 3.45 kb to 61.07 kb, were predicted with PhiSpy (v4.2.6). The presence of these mobile genetic elements suggests that horizontal gene transfer may have played a significant role in the strain’s evolution and environmental adaptation.

To further confirm the phylogenetic position of strain TRM82367, annotated genes with an average nucleotide identity (ANI) greater than 95% and alignment scores above 0.5 were selected based on the Genome Taxonomy Database (GTDB). Combined with its position in the reference phylogenetic tree, the analysis demonstrated that TRM82367 shares an ANI value of 98.11% with *Bacillus velezensis* CR-502, providing additional genomic evidence to support its taxonomic assignment as *Bacillus velezensis*.

The complete genome sequence data has been deposited in the GenBank database under the accession number SUB15732899.

**Figure 3:**
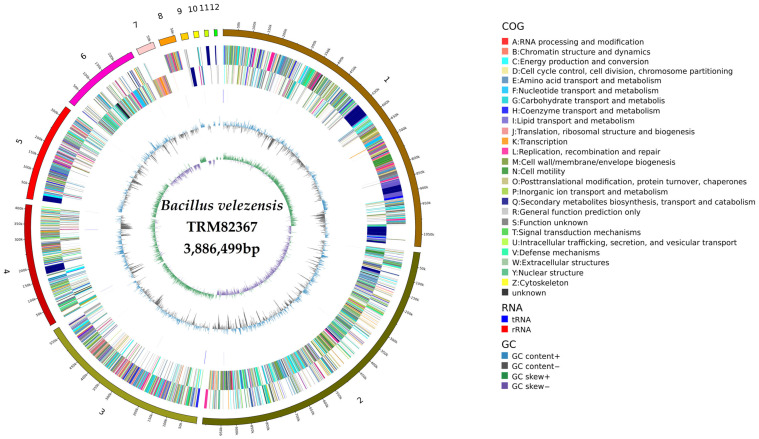
Circos circular map of the TRM82367 genome.

Integrating the results of genome assembly and annotation, a comprehensive circular genome map of TRM82367 was generated using Circos (Figure 3). From the outermost to the innermost ring, the map illustrates the following information: chromosome scale; coding sequences on the forward and reverse strands, color-coded according to their COG functional categories; genomic locations of tRNAs and rRNAs; GC content; GC skew; and the strain designation with genome size at the center.

#### 3.3.2. Prediction of Secondary Metabolite Gene Clusters in *Bacillus velezensis* TRM82367

Analysis via the antiSMASH platform -3-5 against the MiBIG database enabled the prediction of 15 putative secondary metabolite biosynthetic gene clusters (BGCs) within the genome of strain TRM82367 (Table 3). Among these, 10 BGCs exhibited significant similarity to known reference clusters. A total of 6 gene clusters were closely associated with the biosynthesis of nonribosomal peptides (NRPs), specifically clusters 5, 7, 8, 9, 6, and 14. Functional annotation suggested that Cluster 5, Cluster 7, and Cluster 8 are potentially involved in the synthesis of diverse antimicrobial compounds. Cluster 9 was identified as responsible for surfactin production, while Cluster 6 and Cluster 14 were predicted to direct the synthesis of fengycin. Cluster 15 did not yield a definitive prediction outcome.

**Figure 4:**
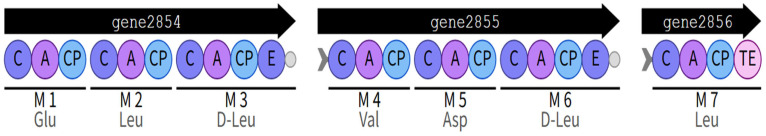
Domain structure of Cluster 9 predicted by antiSMASH in TRM82367.

Further analysis of the modular architecture of Cluster 9 indicated that the surfactin molecule is assembled from seven amino acid residues: glutamate (Glu), leucine (Leu), D-leucine (D-Leu), valine (Val), aspartate (Asp), D-leucine (D-Leu), and leucine (Leu) (Figure 4). Surfactins are characterized by a cyclic lipopeptide structure, comprising a heptapeptide ring linked to a β-hydroxy fatty acid chain. Consistent with known surfactin structural conservancy [22], four amino acid positions were invariant in the strain TRM82367 product: the first residue was Glu, the third was D-Leu, the fifth was Asp, and the sixth was D-Leu. The remaining positions are known to be variable among different surfactin homologs.

### 3.4. Identification of Lipopeptide Components in the Crude Extract

Crude extracts were obtained via hydrochloric acid precipitation of the fermentation supernatant of strain TRM82367 according to the methods described in Section 2.6 and Section 2.7. Liquid chromatography–mass spectrometry (LC-MS) was used to analyze the lipopeptide components.

**Figure 5:**
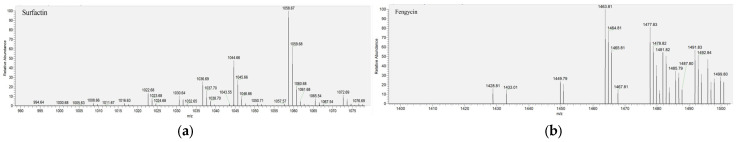
MS1 spectra of the lipopeptides isolated from the crude extract: (**a**) surfactin homologues; (**b**) fengycin homologues, acquired in positive ion mode.

Mass spectrometric analysis in positive ion mode revealed that the crude extract contained homologs of surfactin and fengycin. Specifically, a series of signals with *m*/*z* values of 994, 1008, 1022, 1036, and 1050 were detected for the surfactin homolog series, corresponding to homologs with different fatty acid chain lengths (Figure 5a). For the fengycin homolog series, ion peaks with *m*/*z* values of 1463, 1477, 1491, and 1505 were observed [23] (Figure 5b).

**Figure 6:**
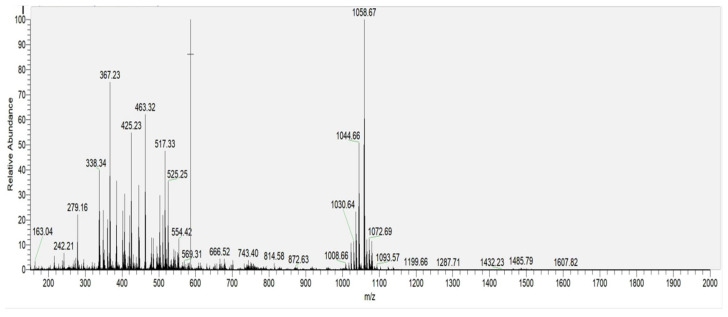
MS1 spectrum of the crude extract acquired in positive ion mode.

As shown in Figure 6, among all detected lipopeptide components, the signal intensity of surfactin homologs was significantly the highest, indicating that surfactin is the main component of the crude extract.

### 3.5. Determination of Insecticidal Activity of the Crude Extract

**Figure 7:**
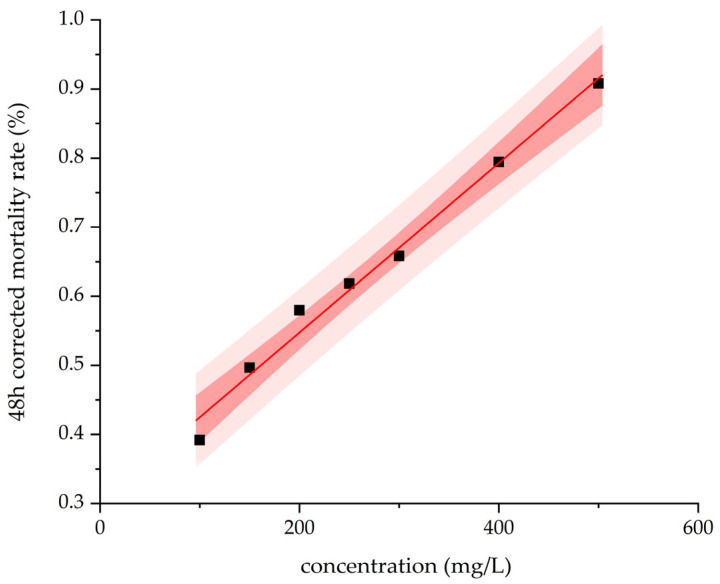
The toxic activity of crude extract against cotton aphids at different concentrations (Dark red and light red are used to depict the 95% confidence interval and the prediction interval, respectively).

The stock solution was diluted with 0.5% Tween-80 to concentrations of 100, 150, 200, 250, 300, 400, and 500 mg/L for aphicidal bioassays. The corresponding 48 h corrected mortality rates of cotton aphids were 25.14%, 38.05%, 50.09%, 53.03%, 57.96%, 74.68%, and 88.67% (Figure 7), respectively. Probit regression analysis of the concentration–mortality relationship yielded the toxicity regression equation Y = 2.47X − 5.72, where Y is the probit-transformed mortality and X is the logarithm of concentration. The goodness-of-fit test (Pearson chi-square) indicated no significant lack of fit (χ^2^ = 3.554, df = 5, *p* = 0.615). The median lethal concentration (LC_50_) and the 95% lethal concentration (LC_95_) were determined to be 207.616 mg/L and 1004.673 mg/L, respectively.

### 3.6. Isolation and Identification of Insecticidal Active Substances

The crude extract was fractionated on an ODS column using a stepwise gradient elution with methanol-water mixtures at concentrations of 10%, 30%, 50%, 70%, 90%, and 100% (*v*/*v*) methanol. Bioassay results indicated that the fractions eluted with 90% and 100% methanol exhibited aphicidal activity against cotton aphids, with the 100% methanol fraction demonstrating the highest efficacy. This most active fraction (100% methanol eluate) was subsequently analyzed using a Thermo Scientific LTQ Orbitrap mass spectrometer for further structural characterization.

**Figure 8:**
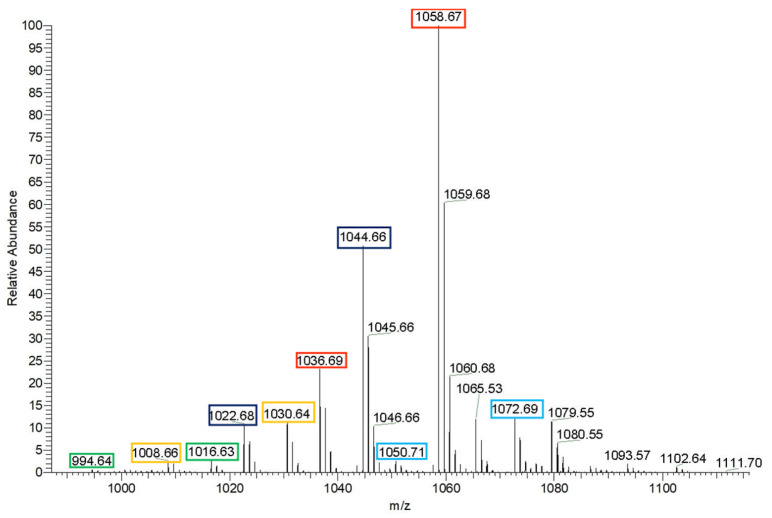
MS1 spectrum of surfactin homologs acquired in positive ion mode.

**Figure 9:**
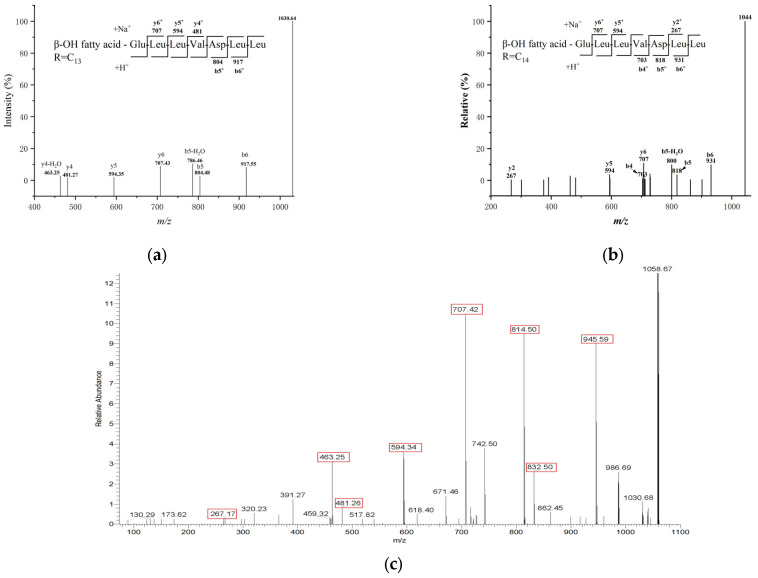
MS2 spectra of C_13_–C_15_ surfactin homologs: (**a**) MS2 spectrum of C_13_-surfactin at *m*/*z* 1030; (**b**) MS2 spectrum of C_14_-surfactin at *m*/*z* 1044; (**c**) MS2 spectrum of C_15_-surfactin at *m*/*z* 1058 (The numbers highlighted in red boxes correspond to the ion fragments discussed in the text).

In the positive ion mode of MS1 analysis, distinct ion peaks corresponding to surfactin homologues were observed (Figure 8). The mass-to-charge ratios (*m*/*z*) were recorded as 994, 1008, 1022, 1036, and 1050, corresponding to [M+H]^+^ ions, along with 1030 (Figure 9a), 1044 (Figure 9b), 1058 (Figure 9c)., and 1072, corresponding to [M+Na]^+^ ions.

Taking the [M+Na]^+^ ion at *m*/*z* 1058 as an example, MS2 analysis revealed a series of fragment ions in the b^+^ series: 1058 → 945 → 832 (with subsequent loss of H_2_O to yield 814) → 714 This fragmentation pattern indicates sequential losses corresponding to a C-terminal Leu–Leu–Asp moiety, where the fragment at *m*/*z* 832 arises from the loss of Leu–Leu, and *m*/*z* 814 results from the loss of Leu–Leu with associated dehydration. The ester linkage is formed between the carboxyl group of the leucine residue and the hydroxyl group in the fatty acid chain of surfactin. Concurrently, a series of y^+^ fragment ions were observed at *m*/*z* 707 → 594 → 481 → 267, suggesting the loss of a Leu–Leu–Val–Asp segment from the peptide chain. Integrating these fragmentation patterns with the gene cluster prediction results, the amino acid sequence of the surfactin ion at *m*/*z* 1058 was determined to be Glu–Leu–Leu–Val–Asp–Leu–Leu.

Furthermore, the consistent mass differences of 14 Da observed among the ions at *m*/*z* 1016, 1030, 1044, 1058, and 1072 [24,25,26] confirm that these represent surfactin homologues with fatty acid chain lengths ranging from C_12_ to C_16_, differing by successive methylene (–CH_2_–) groups in the β-hydroxy fatty acid side chain.

## 4. Discussion

This study successfully isolated and identified a strain of *Bacillus velezensis* TRM82367 from the extreme environment of the Taklimakan Desert, which exhibits potent aphicidal activity against cotton aphids. Using an activity-guided approach combined with LC–MS analysis, we confirmed that the core insecticidal components are a group of lipopeptide surfactin homologs with fatty acid chain lengths ranging from C_12_ to C_16_. This finding not only expands the known functional spectrum of *Bacillus velezensis* but also provides a promising microbial resource and theoretical foundation for its potential application in the biocontrol of cotton aphids.

*Bacillus velezensis* has been the subject of extensive research in agricultural microbiology, though investigations have largely focused on two well-established functional domains: plant disease suppression and growth promotion [27]. In the context of disease control, numerous studies have confirmed its ability to inhibit pathogens such as *Fusarium* [28], *Escherichia coli*, *Staphylococcus aureus* [29], and various phytopathogens [30] through the synthesis of lipopeptides (e.g., fengycin, iturin) and antibacterial polyketides. Regarding plant growth promotion, the bacterium enhances nutrient acquisition by biological nitrogen fixation, phosphate solubilization, and phytohormone secretion [31], aligning closely with the objectives of sustainable agricultural development. However, reports on its direct and highly efficient insecticidal activity against piercing-sucking pests such as cotton aphids remain limited. In this study, the fermentation supernatant of strain TRM82367 achieved a remarkable 48 h corrected mortality rate of 95.23% against cotton aphids (Table 1), and the LC_50_ of its crude surfactin extract was determined to be 207.616 mg/L. Compared with earlier studies, the LC_50_ value obtained here is four times lower [32], indicating notably enhanced insecticidal efficacy. This finding extends the known biological functions of *Bacillus velezensis* from the conventional dual role of “disease suppression–growth promotion” to a tripartite mechanism encompassing “disease suppression–growth promotion–insecticidal activity”, underscoring its significant potential for developing multi-functional microbial pesticides.

Strain TRM82367 was isolated from the Taklimakan Desert, an extreme environment characterized by aridity, significant temperature fluctuations, and nutrient-poor conditions. These harsh conditions may have driven the evolution of unique metabolic adaptations in this strain. Genomic analysis revealed that TRM82367 possesses a rich repertoire of biosynthetic gene clusters (BGCs) for secondary metabolites, with 15 BGCs identified in total (Table 2). These include gene clusters responsible for synthesizing several antimicrobial lipopeptides, such as surfactin, fengycin, and bacillomycin. This substantial secondary metabolic capacity may represent a strategic adaptation to cope with extreme environmental stress and interspecies competition, while simultaneously providing the material basis for its highly effective aphicidal activity. Compared to strains isolated from conventional agricultural soils, TRM82367 demonstrates superior environmental adaptability and higher bioactivity, suggesting that microbial resources from extreme environments hold distinct advantages for developing biocontrol agents tailored for use in challenging agricultural regions, such as arid cotton-growing areas.

Through bioassay-guided fractionation and mass spectrometry identification, this study unambiguously attributes the primary aphicidal activity to a series of surfactin homologues. Surfactin, a cyclic lipopeptide, has historically been investigated predominantly for its antimicrobial, antiviral, and antitumor properties. Here, we quantitatively demonstrate a dose-dependent lethal effect of the crude surfactin extract on cotton aphids, thereby clearly validating the potential application of this class of compounds against piercing-sucking pests. Further LC–MS analysis revealed that the active constituents are a mixture of homologues differing in fatty acid chain length (C_12_–C_16_). Previous studies have indicated that the length of the hydrophobic chain in surfactin is closely related to its bioactivity, with longer chains (e.g., C_14_–C_16_) generally enhancing interaction with hydrophobic regions of biological membranes [33]. In our work, the 100% methanol-eluted fraction, enriched with longer-chain homologues, exhibited the strongest aphicidal efficacy, suggesting that fatty acid chain length may be a critical structural factor governing insecticidal potency. Future studies involving the isolation and activity assessment of individual homologues will be essential to precisely elucidate the underlying structure–activity relationships.

Regarding the aphicidal mechanism of surfactin, two hypotheses were proposed with reference to reports on insecticidal proteins from *Bacillus thuringiensis*: the “intestinal disturbance hypothesis” and the “antifeedant behavior hypothesis.”The “intestinal disturbance hypothesis” suggests that aphids may ingest surfactin during feeding. This compound could affect the stability of intestinal pH or directly damage the structure of intestinal epithelial cell membranes [34,35], thereby interfering with nutrient absorption or causing intestinal dysbiosis. The “antifeedant behavior hypothesis” proposes that surfactin may interfere with the salivary glands of aphids, leading to longer piercing-sucking feeding time and almost complete inhibition of phloem salivary secretion and feeding behavior [36]. These two mechanisms are not mutually exclusive and may act synergistically. Compared with traditional chemical insecticides that target a single site, surfactin’s potential multi-site of action makes it less likely to induce high-level resistance in cotton aphids. This characteristic highlights its advantages as an alternative or rotational insecticide.

In conclusion, *Bacillus velezensis* TRM82367 and the surfactin homologs it produces demonstrate significant potential for developing environmentally friendly biopesticides against cotton aphids. The strain’s core advantages stem from its origin in extreme environments, which likely contributes to its enhanced stress tolerance, the well-defined nature of its active components, and its potentially multi-target mode of action that may slow the development of resistance. Future research will focus on optimizing fermentation and surfactin extraction processes, purifying individual homologs to precisely establish structure-activity relationships, elucidating the molecular mechanisms of action, and ultimately validating field efficacy through practical trials. These efforts are essential to advance this promising candidate from laboratory research to practical application.

## 5. Conclusions

In conclusion, we have screened out the strain *Bacillus velezensis* TRM82367 with strong efficacy against cotton aphids by using the active tracking strategy. The active component against cotton aphids was isolated by means of methanol dissolution and hydrochloric acid precipitation and identified as surfactin. The crude extract of surfactin had a 50% lethal concentration of only 160.82 mg/L, demonstrating its application value in biopesticides. Further optimization of the extraction process, in-depth exploration of the specific mechanisms of each component, and field trials to evaluate the practical application effects are still needed in the future. It is expected that an efficient and environmentally friendly bacterial biopesticide can be successfully developed for the control of cotton aphids.

## Figures and Tables

**Table 1:** Screening results of bacterial strains for aphicidal activity against cotton aphids.

Strain Number	48 h Mortality Rate/%	48 h Corrected Mortality Rate/%
CK	3.5 ± 0.2 f	-
20% imidacloprid suspension	98.27 ± 0.32 a	98.20 ± 0.34 a
TRM82114	56.6 ± 2.17 d	55.03 ± 2.25 c
TRM82357	91.4 ± 1.49 c	87.77 ± 1.49 b
TRM82367	95.4 ± 0.92 b	95.23 ± 0.95 a
TRM82467	52.2 ± 1.95 e	49.83 ± 2.26 d

Note: Data are presented as the mean ± standard deviation (SD). Different lowercase letters within the same column indicate significant differences at the *p* < 0.05 level according to Duncan’s new multiple range test.

**Table 2:** Physiological and biochemical characteristics of strain TRM82367 and *Bacillus velezensis* CR-502.

Test Item	Result	
Glucose	+	+
Sucrose	+	+
Melibiose	−	−
Galactose	−	−
Melanin Production	−	ND
Hydrogen Sulfide Production	+	+
Starch Hydrolysis	+	+
Gelatin Liquefaction	+	+
Milk Coagulation and Peptonization	−	ND
Nitrate reduction	+	+
Citrate Utilization	−	−
Oxidase Activity	+	+
Catalase Activity	+	+
Tween-20	−	−
Tween-60	−	−
Tween-80	−	−

Note: “+” indicates positive reaction; “−” indicates negative reaction; “ND” indicates not detected (data from reference [21] for *Bacillus velezensis* CR-502).

**Table 3:** Prediction results of the secondary metabolite gene cluster of strain TRM82367.

Clusters	Type	Start	End	Most Similar Known Cluster	Similarity
Cluster 1	PKS-like	62,791	104,035	butirosin A;butirosin B	7%
Cluster 2	terpene	186,079	206,819	-	-
Cluster 3	lanthipeptide-class-ii	327,312	356,200	-	-
Cluster 4	transAT-PKS	522,763	610,996	macrolactin H	100%
Cluster 5	transAT-PKS,T3PKS,NRPS	829,687	939,780	Bacillaene	100%
Cluster 6	NRPS,transAT-PKS,betalactone	1,004,411	1,092,066	Fengycin	80%
Cluster 7	NRPS-metallophore,NRPS,RiPP-like	73,613	125,401	bacillibactin	100%
Cluster 8	other	661,714	703,132	bacilysin	100%
Cluster 9	NRPS	149,978	215,385	Surfactin	86%
Cluster 10	transAT-PKS-like	1	46,113	difficidin	53%
Cluster 11	T3PKS	161,099	202,199	-	-
Cluster 12	terpene	265,517	287,400	-	-
Cluster 13	transAT-PKS-like	1	23,693	difficidin	26%
Cluster 14	NRPS	1	12,969	fengycin	13%
Cluster 15	NRPS	1	10,056	-	-

## Data Availability

The original contributions presented in this study are included in the article. Further inquiries can be directed to the corresponding author.
